# Intra-individual variability in ancient *plasmodium* DNA recovery highlights need for enhanced sampling

**DOI:** 10.1038/s41598-024-85038-z

**Published:** 2025-01-04

**Authors:** Alejandro Llanos-Lizcano, Michelle Hämmerle, Alessandra Sperduti, Susanna Sawyer, Brina Zagorc, Kadir Toykan Özdoğan, Meriam Guellil, Olivia Cheronet, Martin Kuhlwilm, Ron Pinhasi, Pere Gelabert

**Affiliations:** 1 Department of Evolutionary Anthropology, University of Vienna, Vienna, Austria; 2 Human Evolution and Archeological Sciences (HEAS), University of Vienna, Vienna, Austria; 3 Facultad de Química y Farmacia, Universidad del Atlántico, Barranquilla, Colombia; 4 Museo delle Civiltà, Roma, Italy; 5 Dipartimento di Archeologia, Asia, Africa e Mediterraneo, Università L’Orientale, Napoli, Italy; 6 Department of History and Art History, Utrecht University, Utrecht, Netherlands

**Keywords:** Evolutionary biology, Archaeology, Population genetics, Pathogens

## Abstract

Malaria has been a leading cause of death in human populations for centuries and remains a major public health challenge in African countries, especially affecting children. Among the five *Plasmodium* species infecting humans, *Plasmodium falciparum* is the most lethal. Ancient DNA research has provided key insights into the origins, evolution, and virulence of pathogens that affect humans. However, extensive screening of ancient skeletal remains for *Plasmodium* DNA has shown that such genomic material is rare, with no studies so far addressing potential intra-individual variability. Consequently, the pool of ancient mitochondrial DNA (mtDNA) or genomic sequences for *P. falciparum* is extremely limited, with fewer than 20 ancient sequences available for genetic analysis, and no complete *P. falciparum* mtDNA from Classical antiquity published to date. To investigate intra-individual diversity and genetic origins of *P. falciparum* from the Roman period, we generated 39 sequencing libraries from multiple teeth and two from the femur of a Roman malaria-infected individual. The results revealed considerable variability in *P. falciparum* recovery across different dental samples within the individual, while the femur samples showed no preservation of *Plasmodium* DNA. The reconstructed 43-fold *P. falciparum* mtDNA genome supports the hypothesis of an Indian origin for European *P. falciparum* and suggests mtDNA continuity in Europe over the past 2000 years.

**Supplementary Information:**

The online version contains supplementary material available at 10.1038/s41598-024-85038-z.

## Introduction

Malaria is an infectious disease caused by various Plasmodium species, whereby at least five infect humans. Plas*modium vivax* has the broadest distribution, while *Plasmodium falciparum* is responsible for most malaria-associated deaths. Both *P. vivax* and *P. falciparum* are believed to have originated in Africa around 50,000–60,000 years ago^1,2^ and spread worldwide with complex patterns of migration, probably following human migrations^1,3–8^. Until the 20th century, malaria was globally spread and its widest-known geographical distribution included most of Eurasia and North America^9,10^. Its present-day distribution predominantly spans warmer climates near the equator, covering Africa, parts of the Middle East, Southeast Asia, China, and the Americas. It is estimated that in 2022, approximately 608,000 people died from malaria^11^. Due to this extensive geographic reach and clinical, malaria remains one of the most significant health threats to humans.

It has been hypothesised that both *P. vivax* and *P. falciparum* may have reached Europe during the Neolithic, about 8,500 years ago, due to a combination of favourable parameters, including climatic conditions, increased human population densities, and the presence of a capable *Anopheles* mosquito vector species^4^. However, this claim lacks archaeological or genetic evidence and remains contested^2^. The current oldest evidence of *P. vivax* comes from a German Middle Neolithic individual and the oldest detected case of *P. falciparum* infection dates back to the Iron Age in Austria^5^. The ancient cases detected so far coincid ewith *Anopheles spp.—*endemic regions^4^. Scholars addressing the effects of this endemic disease on societies in antiquity have stressed the dramatic political and economic consequences of malaria, an issue sometimes neglected by historiography^12–14^. Malaria remained endemic in Europe until the 1970s, extending from the Baltic Sea to the Mediterranean^15^. Currently, less than 20 ancient strains of *P. falciparum*,* P. vivax and P. malarie* have been published.

The identification and sequencing of ancient malaria strains present several challenges. Firstly, osteological lesions are not distinctively indicative of infection. Some are broadly suggestive of underlying infections/diseases but can stem from other similarly-presenting pathologies. One example is recent research suggesting the presence of porous lesions with a higher prevalence in malaria-endemic areas^16^. However, it is impossible to confirm the aetiology of these lesions in archaeological skeletal collections. Consequently, like with *Hepatitis B Virus (HBV)*,* Mycobacterium tuberculosis*,* Yersinia pestis*, and other pathogens, the detection of malaria often depends on extensive random sampling of ancient individuals for genetic studies. Secondly, the success in recovering ancient *Plasmodium* genomes from human remains is contingent on the survival of *Plasmodium* DNA in bone and dental tissues. Some studies have suggested the presence of *P. falciparum* DNA in ancient individuals, such as a 5th -century common era (CE) infant from Lugnano in Teverina, Italy^17^, and ancient Egyptian mummies^18^. However, these studies could not conclusively prove the presence of the parasites, an issue that can now potentially be resolved with next-generation sequencing technologies. A recent study comparing the reliability of antigen detection and DNA sequencing in identifying pathogens in ancient skeletal remains found that, despite a limited sample size, paleogenomics methods are the most dependable for this purpose^19^. When soft tissue is available, microscopy remains an alternative as it is possible to observe the parasite inside the infected erythrocytes^20^. A recent study that screened more than 10,000 ancient individuals could only recover less than 20 strains suitable from mtDNA analyses^5^ .

Paleogenomics also helps to determine malaria’s historical impact on societies, revealing selective pressures and human adaptation to the parasite. Recent research has shown that the significant occurrence of an enzyme deficiency (G6PD) providing resistance against *Plasmodium* infection^21^ among present-day Arab populations likely traces back 6000 years before present (BP), aligning with the onset of the Bronze Age in Eastern Arabia. This indicates a possible link between the spread of agriculture and increased resistance to *Plasmodium* infection^22^. However, the global effect of malaria in ancient populations as a selective pressure is contested^23^.

Ancient *P. falciparum* detections are currently restricted to Eurasia. All known ancient samples show genetic affinities to modern Indian strains, which is evidenced by both nuclear and mitochondrial data^5,6,24,25^. Ancient strains of *P. vivax* from Eurasia spanning almost 1,000 years show similarity to known modern American strains suggesting a likely European colonial origin of these lineages^5,6^. However, data on ancient *Plasmodium* species remains sparse, and there is a need to increase the number of *Plasmodium* strains to enhance our understanding of Plasmodium dispersal patterns and affinities in historical contexts, specially within periods linked to extensive mobility through the Mediterranean such as the Roman Period^26^.

To date, no aDNA studies have assessed the differential preservation of *Plasmodium* aDNA across skeletal elements of the same individual. Here, we introduce the first Roman-era full mitochondrial genome sequence (43-fold coverage) of *Plasmodium* falciparum, derived from the individual known as Velia-186 (LV13), previously confirmed to be infected with the pathogen^27^. We demonstrate extensive differences in *Plasmodium* reads yields from different teeth of the same individual suggesting a differential and randomized preservation pattern, which required broad sampling at individual basis to overcome this limitation and recover substantial *Plasmodium* data.

## Results

**Fig. 1:**
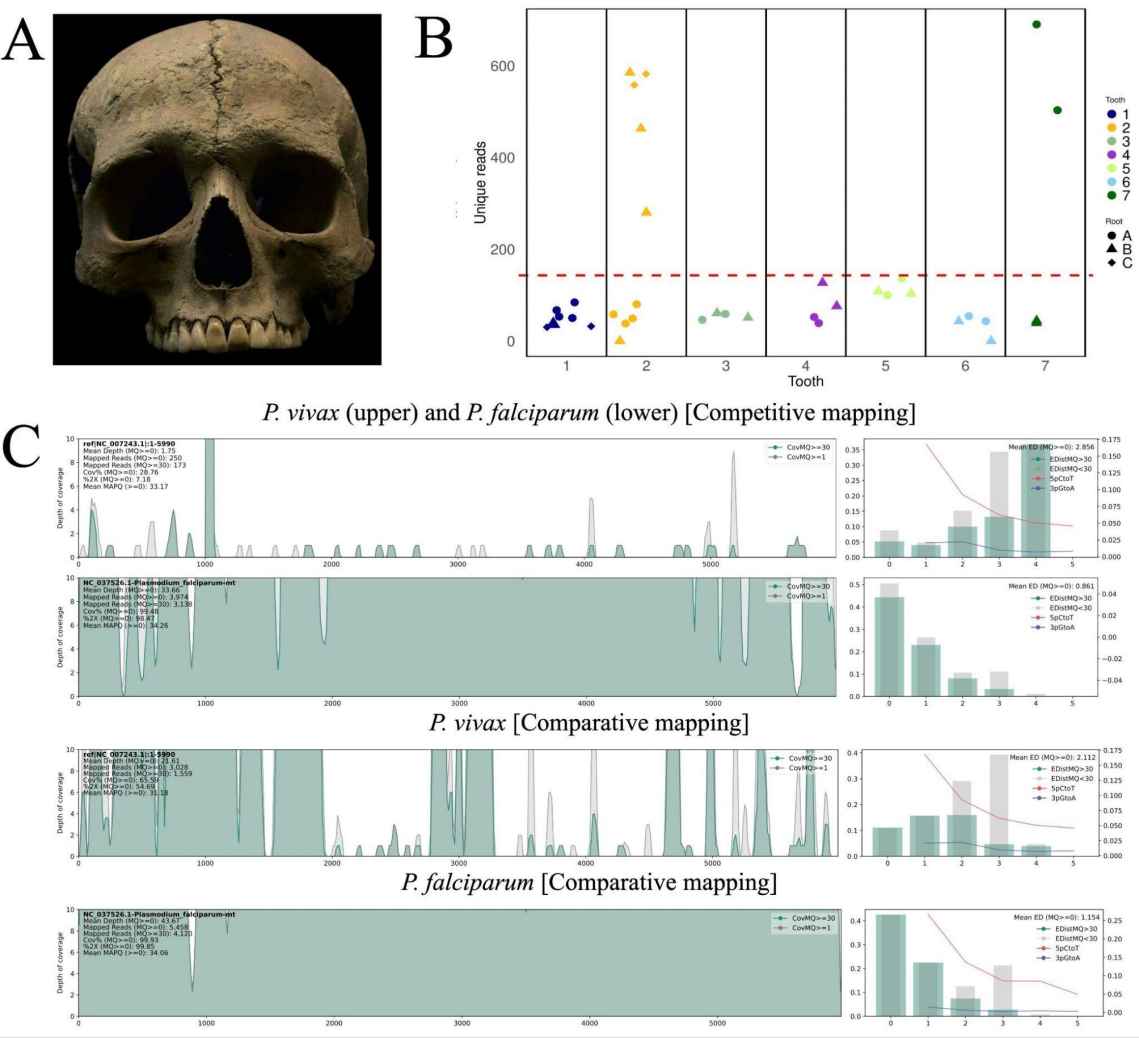
(**A**) Velia-186 skull (male 20–25 years old). Photo by the Museum of Civilizations. (**B**) The number of unique reads mapping to the reference genome K1 [NC_037526] was recovered from each sequencing library. The red line denotes the average amount of reads recovered per library. Samples 2, 3 (Upper right second molar), and 8 (Lower right first molar) contribute the majority of reads. (**C**) Mapping plots to both *P. vivax* and *P. falciparum*. Reads with an MQ of or above 30 are depicted in green. The coverage is shown across the whole mitochondrial genome. The bar plot on the right illustrates the edit distance and the percentage of C -> T mutations at the 5’ end and the G -> A mutations at the 3’end.

In an initial screening, we produced sequencing libraries from both teeth and parts of the femur of individual Velia-186 (Velia, Porta Marina necropolis, I-II cent. CE, Fig. 1A). We targeted teeth based on previous evidence that pathogens are generally well-preserved in dental tissues^28^, and the femoral diaphysis and head, as *P. falciparum* gametocytes commonly mature in human bone marrow^29^. As one of the libraries from the lower left premolar yielded just over 100 unique reads for *P. falciparum* (Table S1C), we decided to test the possibility of differential recovery across the individual’s dentition. For that purpose, 38 DNA libraries from seven teeth were generated by sampling at least two roots per tooth (see Table S1), to asses if different roots from the same tooth would yield different amounts of *Plasmodium*. The libraries were enriched with in-solution baits covering both *P. falciparum* and *P. vivax* mitochondrial genomes and sequenced on an Illumina NextSeq 550, generating paired-end reads with a length of 150 bp. Following preprocessing, quality control, and collapsing, a total of 946 million reads were recovered, with an average of 22 million reads per library (standard deviation (SD) = 1.167 million). The data was then merged for subsequent analysis. To assess whether the recovered reads originated from a co-infection of *P. falciparum* and *P. vivax*, we performed competitive mapping, which maps reads to different reference genomes simultaneously to ascertain which genome the read fits best. Competitive mappings for *P. falciparum* and *P. vivax* resulted in 33.66-fold (Fig. 1C, Table S1) and 1.75-fold (Fig. 1C, Table S1) depth of coverage, respectively, indicating that the vast majority of reads stem from *P. falciparum* and not *P. vivax*. We then performed comparative mapping, which maps to each genome individually to obtain the coverage per species. This mapping resulted in a mean depth of 21.61 for *P. vivax*. However, only 22% of the genome had coverage due to uneven mapping, which indicates mismapping. On the other hand, the mapping to *P. falciparum* showed an even distribution of coverage with an average depth of 43.67-fold (Fig. 1C, Table S1). Based on these results, we deduce that the Velia-186 individual was only infected with *P. falciparum* and exclude the possibility of a co-infection with *P. vivax* as in Ebro-1944 or STR samples. Hence, below, we focus on reads mapped to the *P. falciparum* mtDNA genome.

In our study, following the identification of only *P. falciparum* infection, we proceeded to analyse the *P. falciparum* DNA across various sequencing libraries. This analysis was centred on aligned reads with mapping scores above 30 (Table S1). Notably, we found no reads aligning to *P. falciparum* on the external surfaces of the first right inferior premolar nor in the femoral diaphysis or head (Table S1). On average, each library from the seven teeth produced 108 reads (with a standard deviation of 180 across all 38 libraries), which shows significant diversity. Intriguingly, a mere seven out of the 38 libraries contributed to 72% of the total unique fragments recovered (see Fig. 1B). Moreover, there is a clear uneven preservation pattern between the human and *P. falciparum* ancient DNA (aDNA), where different teeth yielded more unique reads for one or the other (see Fig. 1B and Fig. S2). Additionally, we inspected misincorporation patterns characteristic of aDNA, finding a clear deamination pattern in both 5’ (22%) and 3’ (18%) ends, corroborating an ancient origin of the sequencing data (Fig. 1C). Furthermore, the read-length distribution showed the typical small fragment lengths associated with ancient DNA (Table S1).

We analysed a potential correlation between the dental samples DNA yield and their respective unique Q30 mapped reads but found no significant correlation (R^2^ = 0.02 and *p-value* = 0.9) (Table S3). Further, considering the non-normal distribution of reads, we investigated whether there was greater diversity within the different roots of one tooth or between the teeth. This revealed no significant variations in their medians ((χ2(6) = 11.06, *p-value* = 0.08) (Table S3), suggesting that homogeneity of sequencing yields was observed between the dental samples.

We combined the 5458 mapped reads from all seven teeth, yielding a mitochondrial genome of *P. falciparum* with an average depth of 43-fold. Next, we used BLASTn^30^ to identify any potential sources of contamination. BLASTn classified 4120 filtered reads that we later classified at the family/order level with MEGAN using an LCA algorithm^31^. From these, 3,913 were successfully classified, all in the order of Haemosporida, which confirmed that only *P. falciparum* is present and no contamination from the soil was affecting our samples. Following these results, we decided to use the 5458 *P. falciparum* reads for the downstream analyses.

We used the 5458 *P. falciparum* reads to generate a consensus sequence with ANGSD (version 0.941) and obtained a mitochondrial genome with 99.1% of the sequence covered. The consensus sequence has three gaps, with a size of 59 (858–917), 20 (1151–1171), and 9 (3495–3504) base pairs, respectively. The SNPs in our consensus sequence were inspected individually in IGV (v2_16.0) to verify and validate their presence. Our high-coverage genome consensus sequence has seven mutations compared to the reference strain mitochondrial genome (74, A -> T; 276 G -> A; 725, C -> T; 772, T -> C; 2172, T -> C; 2763, C -> T; 3938, A -> T) all supported by at least ten reads (see Table S2). Four of the seven mutations are located in coding regions of the mtDNA (Table S2). However, none of these are non-synonymous using the Apollo^32^ annotation tool accessed through PlasmoDB (v. 68)^33^. More details about the mutations are presented in Supplementary Table S2.

**Fig. 2:**
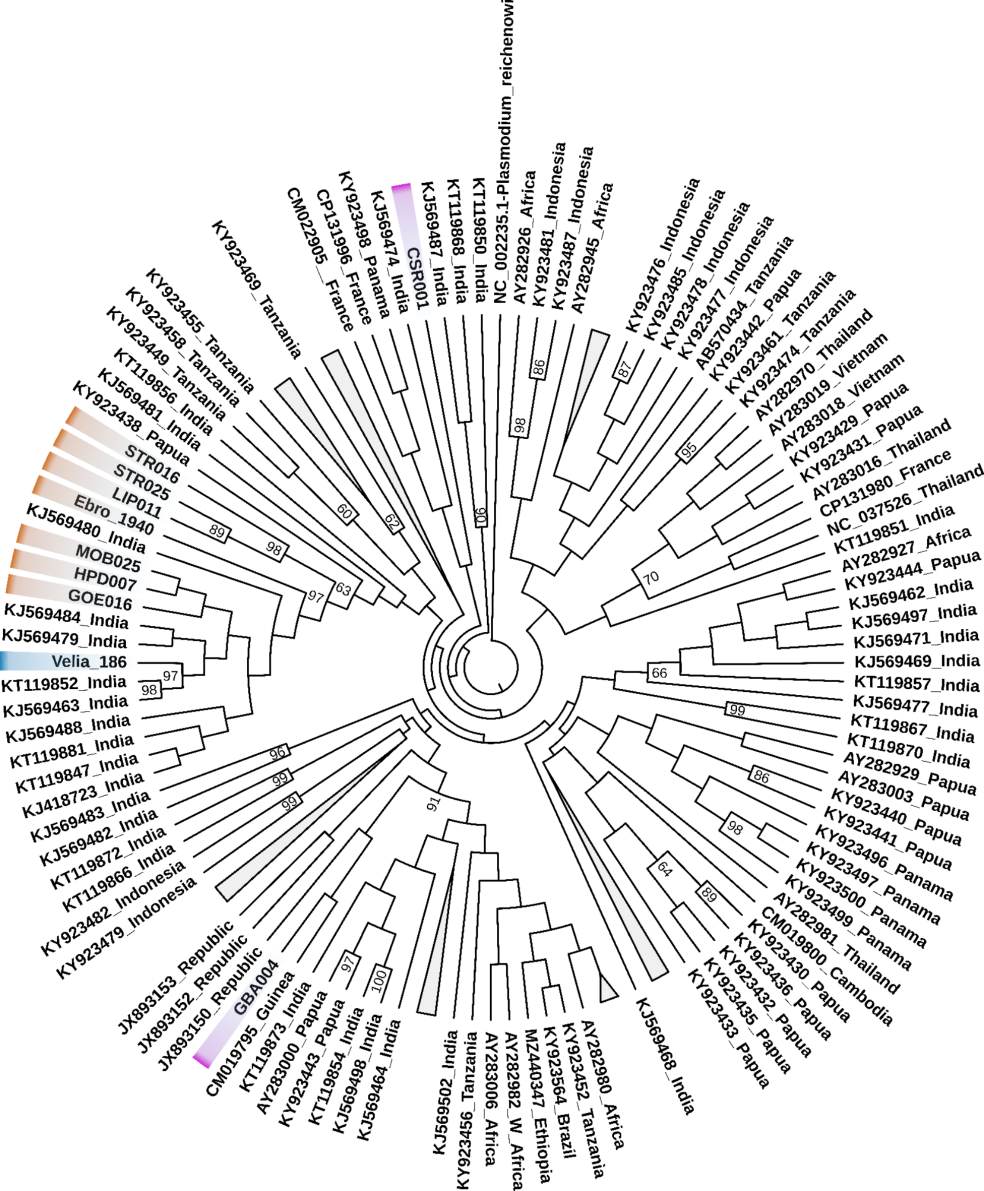
*P. falciparum* mtDNA maximum likelihood phylogeny. It is observable that the* P. falciparum* from Velia-186 (in blue) clusters with Indian strains and previously published ancient* P. falciparum* strains (in orange). Additional ancient strains of* P. falciparum* are in purple. The tree was visualised with TreeViewer^37^. We present a rooted phylogeny without branch lengths, and numbers represent bootstrap values.

To elucidate how our *P. falciparum* consensus sequence compares to other *P. falciparum* genomes, we downloaded 345 mitochondrial genomes of *P. falciparum* strains from NCBI, representing all presently known *P. falciparum*. (see Table S4). First, we observed that only two substitutions, at positions 2,172 (T > C) and 3938 (A > T), were not present in any *P. falciparum* strain of the dataset. Next, we performed a maximum likelihood tree using a multiple sequence alignment, including our ancient consensus sequence (*n* = 345). Although some clades cluster geographically, we observed that the current mtDNA genetic diversity does not exclusively reflect a geographical distribution, as previously reported^34^. In the phylogenetic tree, Velia-186 clusters exclusively with strains that are currently found in India as well as some ancient strains from Spain, France, Austria, Germany, Belgium, and Taiwan dated to different periods, with a 97 bootstrap support (Fig. 2 and S1). Out of the seven SNPs described above, mutations 276 (G > A), 725 (C > T) and 2763 (C > T) have been observed in present-day strains from different locations in India^35^. These SNPs are characteristic of the Indian subclade called PfIndia, described by Tyagi et al., 2014^35^, which further supports the phylogenetic clustering of the Velia-186 sequence close to the Indian strains. Further, the three mutations also exist in Ebro-1944, GOE016, LIP011, STR025, STR016 and MOB025 indicating genetic similarity between ancient European samples.

We tested the capacity to recover nuclear DNA from the seven best libraries by shotgun sequencing (Table S1D). The overall endogenous content is very low (below 0.01%). Out of 50.000.000 sequenced reads, we only recovered 1931 unique ones belonging to *P. falciparum.* Assuming the current complexity would enable it, we estimated it would require more than 16.000 million sequencing reads to target a 1X *P. falciparum* genome, the prohibitive cost of which makes it impossible.

We also aligned the sequencing reads against the human genome (hg19), including the mtDNA sequence (rCRS). We could recover 2,141,320 reads by combining all the libraries; 53,022 reads are aligned to the human rCRS genome. We observe differences in human DNA preservation per tooth, but these do not coincide with the preservation of *Plasmodium* DNA, as most reads are from tooth 6 (Fig. S2). The recovered reads also show consistent signals of deamination due to age (Fig. S3). We recovered an 1169X mtDNA human sequence and identified that the individual Velia 186 belongs to haplogroup T2b7a (88%), which is already identified in MBA individuals from the Levant^36^.

## Discussion

Implementing next-generation sequencing (NGS) in paleogenomics has enabled the detailed study of microbial agents’ genomic diversity, evolutionary trajectories, and pathogenicity, moving beyond merely detecting their presence in ancient remains^28^. Detecting *Plasmodium* infections in ancient human remains is still^19^ particularly challenging due to limited genomic material and degraded samples^27^, making it difficult to obtain comprehensive mtDNA or genomic data for pre-20th century *P. falciparum.* Building on prior findings that Velia-186 was infected with *Plasmodium*^27^, this study aimed to sequence the complete mtDNA to infer its genetic affinities and assess the differential preservation of *Plasmodium* DNA across skeletal elements. Here, we show that these challenges persist beyond identifying infected individuals through extensive sampling, highlighting the importance of selecting appropriate skeletal elements for analysis. Based on our results, we recommend sampling multiple teeth per individual to improve the likelihood of successful *P. falciparum* recovery in case of poor DNA recovery after initial sampling and identification. Our results highlight how the processing of single samples do not reflect the overall preservation for target genomes across an individual. This study thus provides valuable insights into refining sampling strategies to optimize recovery of *P. falciparum* from ancient skeletal remains.

After analysing 41 libraries from the same individual, we have uncovered significant intra-individual variability in the presence of *Plasmodium* within a single individual or even tooth. This finding underscores the importance of employing a sampling strategy that includes multiple samples from the same individual for effective pathogen detection rather than relying solely on a single sample. Notably, After multiple sampling from dental pieces, most *Plasmodium* reads were concentrated in only seven libraries out of a total of 39. This study highlights the need for a larger sample to potentially unveil the finer preservation patterns of *Plasmodium* DNA. Additionally, our observations indicate that the quantity of *Plasmodium* DNA detected does not appear to correlate with the amount of host DNA present. For instance, tooth 6 (Lower molar left 3) yielded predominantly human DNA but minimal *Plasmodium* DNA (it yielded 44% of the entire human reads but below the 2% of *Plasmodium* ones). Our results suggest that the varying levels of *Plasmodium* DNA in our sample set may be due to differences in DNA preservation between teeth or to variations in parasite levels in the bloodstream peri-mortem, which could affect the likelihood of obtaining sufficient material from each tooth^38^. This underscores the challenge of recovering nuclear DNA from *Plasmodium* in aDNA samples, suggesting that future sequencing efforts might require extensive sampling to be successful.

Our findings provide definitive evidence for the continuity of *P. falciparum* in Europe during ancient times^5^. Remarkably, the mitochondrial genome of Velia-186, originating from an individual nearly two millennia old, shows a close phylogenetic relationship with almost all other ancient *P. falciparum* strains, especially from Europe and Asia spanning from the Neolithic to colonial times. This connection suggests the genetic continuity of the parasite in Europe over the past 2000 years. In a previous study on the same individual based on only a few sequenced fragments and low depth of coverage, Marciniak et al. (2016) reported 21 mutations between the ancient genome and the mitochondrial *P. falciparum* reference genome. Our high-coverage consensus sequence has seven mutations compared to the reference mitochondrial genome. When comparing our high-coverage SNP call with previously reported mutations for LV-13^27^ we could only verify the presence of two of the mutations (2763, C -> T; 3938, A -> T). Additionally, compared to LV-13, this genome carries five novel mutations (74, A -> T; 276 G -> A; 725, C -> T; 772, T -> C; 2172, T -> C), which are all supported by at least ten reads (see Table S2). The previously reported higher number of mutations, reported for LV-13, is probably the result of the low coverage of degraded DNA, which could have impacted the SNP call. Remarkably the report of two undescribed mutations, evidences that the diversity of past strains is still not fully described. Although we lack nuclear data, the published evidence strongly indicates that the recovered strain belongs to the endemic ancient *P. falciparum* of Eurasia. Future extensive sampling of the region over time could illuminate regional differences, enhance our understanding of the pathogen’s fine structure in antiquity, and relate it to human movements and health conditions.

## Materials and methods

### Archaeological context

We sampled seven dental pieces from Individual Velia-186. Several bioarchaeological analyses have been carried out on individuals from this cemetery^39–41^, and *P. falciparum* was identified genetically in the individual we selected for downstream analyses^27^. This sample is from the 1st -2nd centuries CE.

Velia is located on a peninsula on the Tyrrhenian coast 112 km southeast of Naples and was incorporated into the Roman territory in the third century BCE. It became a port city utilised for the shipment of goods, boat maintenance, fish processing, and arboriculture. Subsistence strategies also included cultivation in the hinterland and well-watered intramural areas. The cemetery of Porta Marina (1st -2nd centuries CE) was investigated by Fiammenghi (2003) and led to the identification of approximately 330 burials (mostly inhumations)^42–44^. The human skeletal material is entrusted for anthropological study to the Bioarchaeology Service of the Museum of Civilizations based in Rome to reconstruct the funerary rituals and describe the demographic and bio-social characteristics of the ancient inhabitants of Velia within an interpretative framework guided by historical-archaeological evidence.

### Experimental model and subject details

Velia-186 is represented by a complete and well-preserved skeleton. Morphological and morphometric analyses led to a diagnosis of a young adult male (20–25 years). The values of oxygen and strontium isotopes indicate that the individual was likely born and raised in Velia^45,46^. The skeleton shows a robust morphology with mildly developed muscle attachments. The estimated living height (by Pearson regression formulas on the femur maximum length) is 166.9 cm, slightly above the average of the VELIA male series (164.7 ± 4.8 cm). The individual presents *cribra orbitalia* on both orbital roofs. The lesions (Type 3, according to Stuart-Macadam 1991^47^ are in a remodelling phase^40^.

### Laboratory procedures

100 mg was ground from multiple locations of different teeth and bone material including dentition pieces and other anatomical locations (Table S1A and S1C), and all procedures were done with permission from Museo delle Civilta. DNA was extracted from bone powder in the Ancient DNA (aDNA) Laboratory at the University of Vienna following detailed protocols adapted to aDNA^48^, and single-stranded libraries were prepared^49^. Libraries were enriched with an in-solution capture designed by Daicel Arbor Biosciences (https://arborbiosci.com/products/targeted-ngs/mybaits-custom-kits/mybaits-custom-dna-seq/*)* following the manufacturer manual. The solution kit included baits targeting the mtDNA sequences of both *P. falciparum* and *P. vivax.* Libraries were pooled in 20 *µl* and sequenced 2 × 150 PE on NextSeq 550 at the Polo d’Innovazione di Genomica Genetica e Biologia (Siena, Italy).

#### Bioinformatics

Sequenced reads were clipped with cutadapt 4.5^50^, removing Illumina adapters and base quality < 30 and read length < 30, and later collapsed using PEAR version 0.9.11^51^, requiring a minimum overlap of 11 bp and a minimum length of 30 bp. Filtered collapsed reads were aligned with a competitive mapping of *P. vivax*,* P. falciparum* and human mitochondrion (Salvador 1 [ID: NC_007243.1], K1[ID: NC_037526] and H. sapiens [ID: NC_012920.1], respectively) using Burrows-Wheeler Aligner (BWA) 0.7.17 aln^52^, whereby aln stands for the alignment mode and enables mapping of short DNA sequences. Also we disabledseeding to allow a higher sensitivity of aDNA reads^53^, gap open penalty of 2 and edit distance of 0.04. Next, the mapped reads were deduplicated using the MarkDuplicates program in Picard-tools 3.1.1^54^ and low mapping-quality reads were removed using the flag -q 30 in Samtools 1.9^55^. We calculated the deamination pattern using MapDamage^56^; mapping plots were created using aDNA-BAMplotter^57^. We used this competitive mapping between *P. falciparum and P. vivax* to determine the possible presence of co-infection. We used Samtools 1.9 ^52^ to convert the bam files into fasta files that were screened with BLASTn^30^ 2.15.0 using the whole NCBI nt database (2023-08-17). The results were uploaded to MEGAN 6.25.9^31^ and processed with the LCA algorithm.

A consensus sequence was obtained using ANGSD (v0.941)^58^, which used the majority of calls, and excluding sites with coverage lower than 5, all the SNPs were fixed. The detected mutations were examined visually with IGV^59^. The consensus sequence was aligned with multiple *P. falciparum* sequences^6–8,35,60–63^ with MAFFT (v7.520)^64^. For the phylogeny, the alignment was filtered by positions shared by 90% coverage to eliminate incorrectly assembled regions from the analysed sequences, which left 5967 sites, including 230 variable sites. The TPM2u + F + I substitution model was chosen with IQTree2 (v 2.2.6) using the ModelFinder algorithm. Due to alignments containing the whole sequence with variant and invariant sites, no ascertainment bias corrections were needed. We constructed a Maximum likelihood tree with 1000 nonparametric bootstrap replicates. The tree was visualized via *TreeViewer*^37^.

### Human mtDNA analyses

We aligned the reads against the human genome (hg19), including the mtDNA sequence (rCRS), using the same procedures previously described. We recovered the consensus sequence using ANGSD^58^ and identified the haplogroup using Haplogrep 3.2.2.1^65^.

## Electronic supplementary material

Below is the link to the electronic supplementary material.

Supplementary Material 1

Supplementary Material 1

## Data Availability

Sequencing data and the filtered sequences are available at the European Nucleotide Archive (ENA) under the accession number PRJEB72667.
