# Supplementary material for: Intra-individual variability in ancient plasmodium DNA recovery highlights need for enhanced sampling
Source: Sci Rep. 2025 Jan 4;15:757. doi: 10.1038/s41598-024-85038-z (PMC11700196; doi:10.1038/s41598-024-85038-z)
Supplement: Supplementary file 1 — Supplementary Material 1 [file 41598_2024_85038_MOESM1_ESM.docx]

**Complete mitochondrial genome of a Roman-era *Plasmodium falciparum***

**Supplementary Figures**


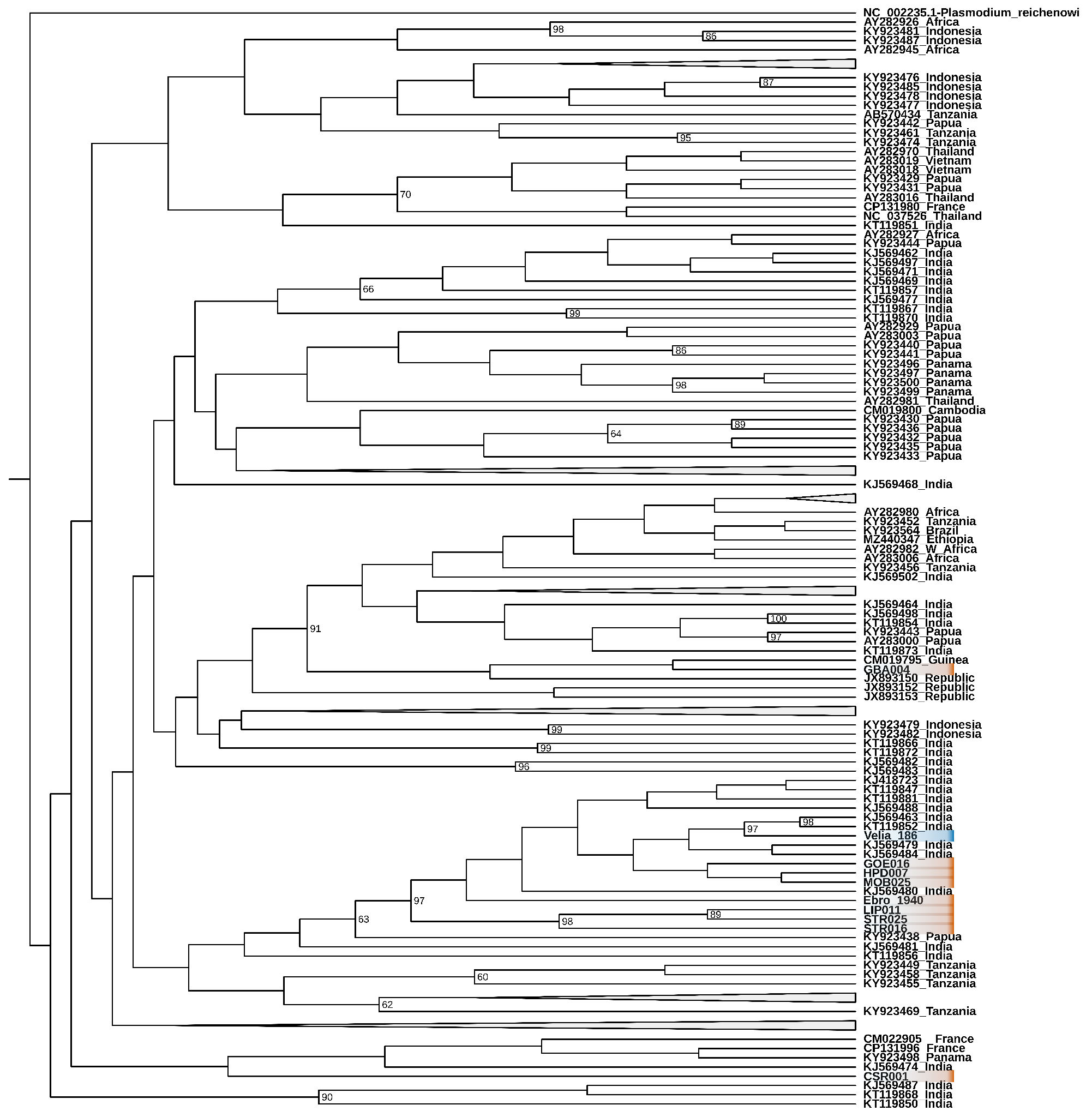


**Supplementary Figure 1:** *P. falciparum* mtDNA Maximum Likelihood phylogeny. The sample sequenced in this study is written in Blue (Velia-186), and the other ancient mtDNA European sequence are in red. Additional ancient strains of P. falciparum are in purple. Numbers in branches denote bootstrap values.


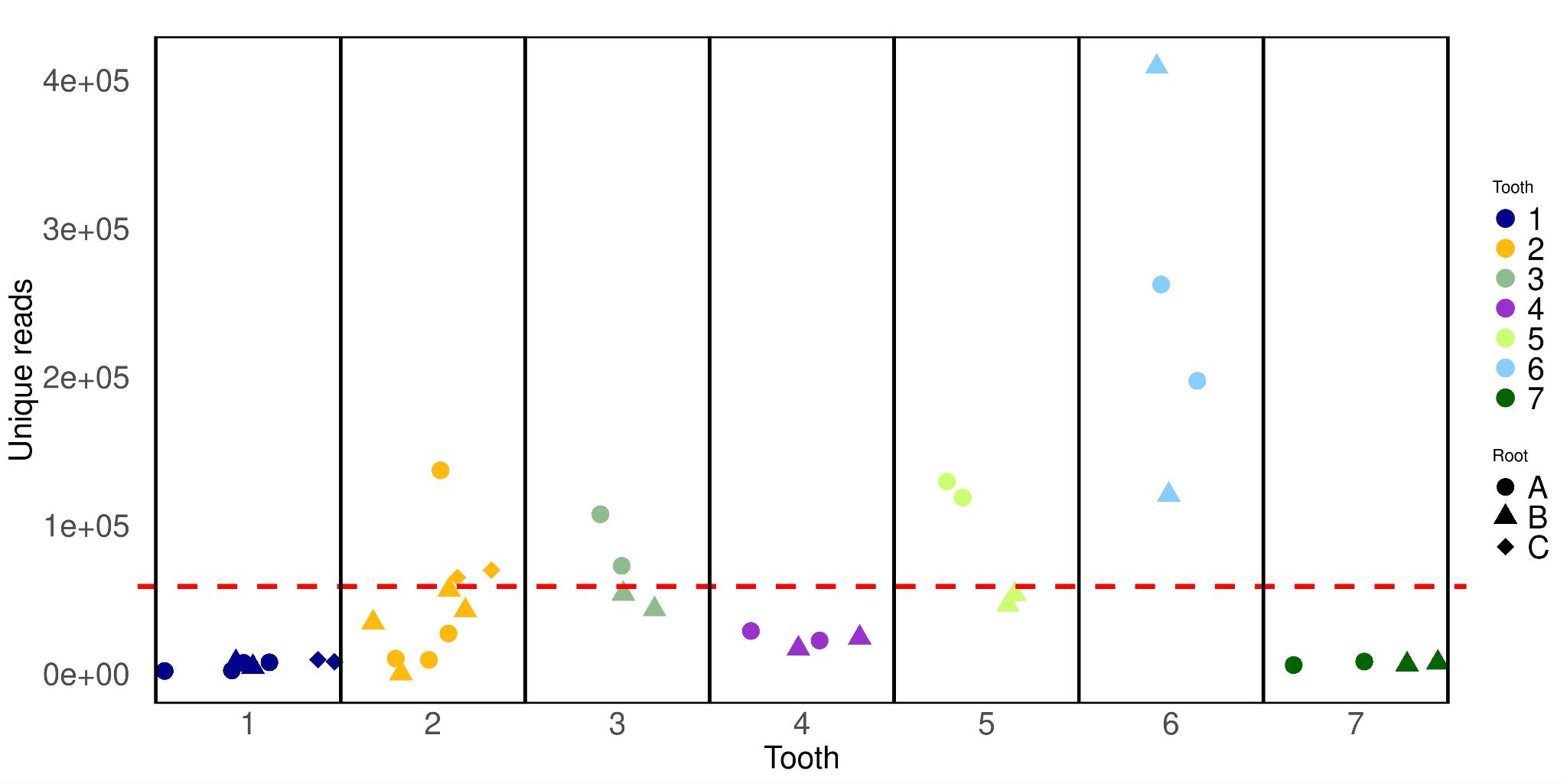


**Supplementary Figure 2:** Human DNA reads recovered per each of the libraries, the red dots line represents the average of the whole individual.


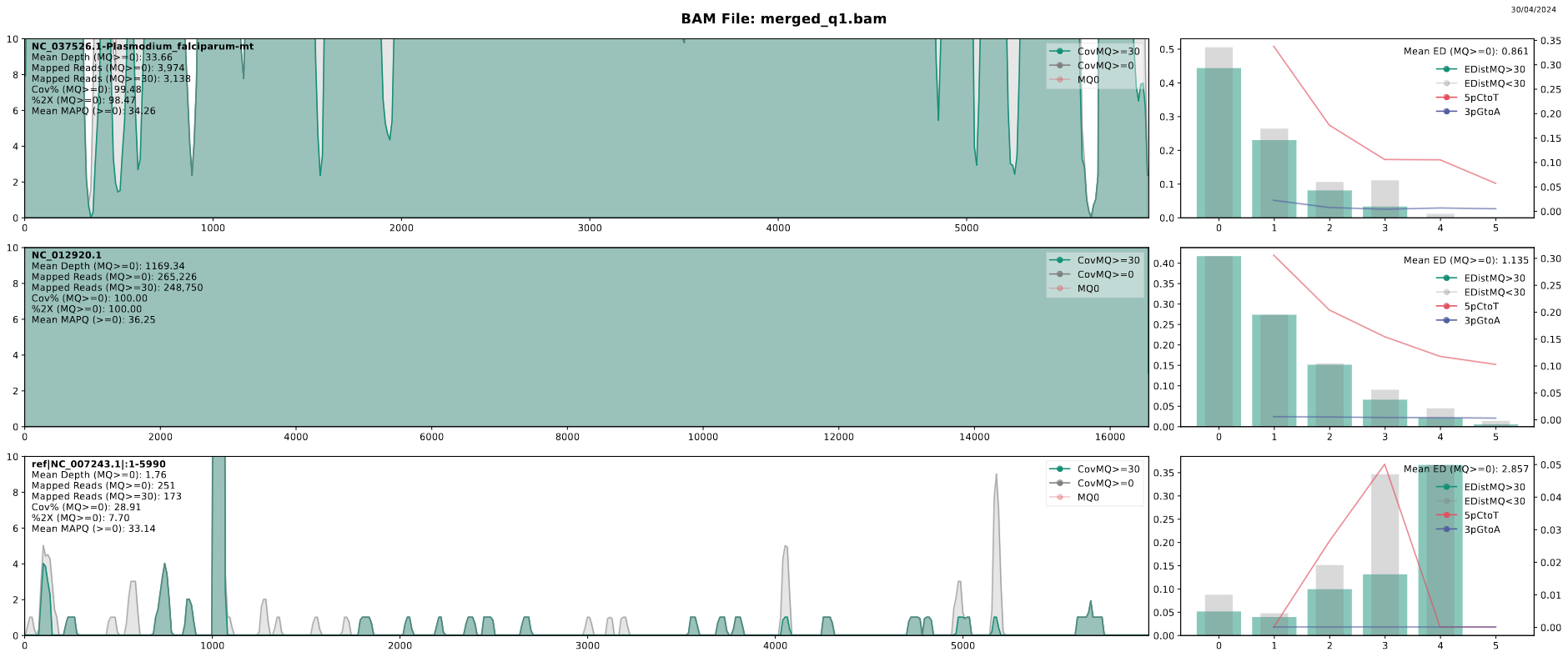


**Supplementary Figure 3:** Mapping plots to A) *P. falciparum, B) Homo sapiens* mtDNA and C) *P. vivax*. Reads with an MQ of or above 30 are depicted in green. The coverage is shown across the whole mitochondrial genome. The bar plot on the right depicts the edit distance and the percentage of C -> T mutations at the 5`end and the G -> A mutations at the 3’end.
